# The Cigarette

**Published:** 1886-07

**Authors:** C. H. Bushong

**Affiliations:** 690 Hudson St.


### THE CIGARETTE.

BY C. H. BUSHONG, M. D.

The disagreeable custom of cigarette smoking is constantly increasing. It is therefore proper that we should make inquiry concerning its effects upon those that may acquire it.

We will merely hint at some of the objections to its use that are based on grounds of sentiment only, before going on to the more practical points of its effects on the health and chances of longevity of its *habitues*. The undignified figure cut by a grown man smoking a cigarette is evident to any one who has seen an example—also the disgusting picture of a small boy who feels that his smoking makes him very tall. The smoke from these little pests is offensive to all non-smokers, while that of a cigar may be rather agreeable to some. Then the money expended for this useless habit foots up at no small amount.

In these days of a general knowledge of physiology and hygiene, it is not necessary to describe the structure of the interior of the lungs, or the mechanism through which the air reaches their innermost cavities.

The objections to the habit of smoking are more directly applicable to the cigarette smoker, because he usually inhales the smoke. This brings it in direct contact with the mucous membrane lining interior of the lungs and tubes leading to them. The cigarette being small, is used more of the time, and thus these delicate tissues are kept in contact with this heated smoke a greater proportion of the time than is the case with that of those who smoke cigars or a pipe. The consequences will be evident to any one who knows the effect of warm smoke on raw flesh. Meat is *smoked* in this way. Remembering this, it is easy to imagine the condition of the interior of the lungs of a smoke inhaler. The membrane is tanned and hardened, the secretions increased, and swelling of the membrane ensues. The integrity of the the lungs is materially interfered with and their power to resist disease processes is much lessened.

When we learn that more than one-eighth of the deaths in the U.

S. from all causes is due to disease of the lungs, we feel that our apprehensions from the extent and increase of the cigarette smoking habit are well founded. In fact any custom that interferes with the healthfulness of the lungs is a source of danger to the race.

The cigarette is especial cause for alarm because the greater number of its users are so young, and in the most susceptible period of life—that of growth and developement. Forces acting at this time exert an influence over the whole after life. It is in this period that the germs of later lung trouble are usually planted, and the unfortunate victim finds himself afflicted with "consumption" either in a rapidly fatal form, or, if discovered in time, and rationally and persistently treated, he must eke out a precarious existence. He must be constantly on his guard against colds, etc., and is ever after more or less, an invalid. Such a man is a constant source of anxiety to himself and his friends ; his ability for work or enjoyment being very much lessened by his disease. When it is understood that few cases of "consumption" exist that are not directly traceable to a violation of some hygienic law, the importance of carefully watching for and calling early attention to every such violation will be appreciated. A general diffusion of knowledge of the requirements of how to live properly will do much to free us from this terrible scourge.

The inhaling of smoke of any kind helps to weaken the lungs and predisposes them to disease. When this fact is generally known and its importance appreciated, parents and guardians will cease to consider the cigarette "one stage of a boy's developement," but will, knowing the danger, unsparingly condemn and prohibit its use.

The moral effects of this habit are due to the nervousness that is caused by it. When deprived of his cigarette, the cigarette smoker is irritable and fretful. He cannot remain quiet. This is due to the opium that is in almost all brands, and the nicotine with which his system soon becomes soaked, the need of keeping up a supply of these soon makes him "a bundle of nerves." His capacity for work is diminished and his powers of endurance are less. His mental strength is impaired, his judgment less clear and his conscience blunted.

The cigarette smoker also suffers in common with all who indulge a habit, from the weakening effects of that indulgence. This favors the growth of selfishness and a diminution of the powers of resistance, which makes him less capable of resisting other temptations.

It is best for your health of body and mind not to smoke, and especially bad for growing boys to smoke cigarettes.

690 Hudson St.

